# Early neonatal mortality is modulated by gestational age, birthweight and fetal heart rate abnormalities in the low resource setting in Tanzania – a five year review 2015–2019

**DOI:** 10.1186/s12887-022-03385-0

**Published:** 2022-05-27

**Authors:** Aisa Shayo, Pendo Mlay, Emily Ahn, Hussein Kidanto, Michael Espiritu, Jeffrey Perlman

**Affiliations:** 1 Department of Pediatrics, Kilimanjaro Christian Medical University College, Moshi, Tanzania; 2 Department of Obstetrics and Gynecology, Kilimanjaro Christian Medical University College, Moshi, Tanzania; 3 Present address: Division of Newborn Medicine, Department of Pediatrics, Weill Cornell Medicine, New York Presbyterian Hospital, 1283 York Avenue, Box 106, New York, NY 10065 USA; 4 Department of Obstetrics and Gynecology, Aga Khan University, Dar Campus, Dar es Salaam, Tanzania

**Keywords:** Neonatal mortality, Fetal heart rate abnormalities, Birth asphyxia, Helping babies breathe, Bag mask ventilation, Hypothermia

## Abstract

**Background:**

Early Neonatal mortality (ENM) (< 7 days) remains a significant problem in low resource settings. Birth asphyxia (BA), prematurity and presumed infection contribute significantly to ENM. The study objectives were to determine: first, the overall ENM rate as well as yearly ENM rate (ENMR) from 2015 to 2019; second, the influence of decreasing GA (< 37 weeks) and BW (< 2500 g) on ENM; third, the contribution of intrapartum and delivery room factors and in particular fetal heart rate abnormalities (FHRT) to ENM; and fourth, the Fresh Still Birth Rates (FSB) rates over the same time period.

**Methods:**

Retrospective cohort study undertaken in a zonal referral teaching hospital located in Northern Tanzania. Labor and delivery room data were obtained from 2015 to 2019 and included BW, GA, fetal heart rate (FHRT) abnormalities, bag mask ventilation (BMV) during resuscitation, initial temperature, and antenatal steroids use. Abnormal outcome was ENM < 7 days. Analysis included t tests, odds ratios (OR), and multivariate regression analysis.

**Results:**

The overall early neonatal mortality rate (ENMR) was 18/1000 livebirths over the 5 years and did not change significantly comparing 2015 to 2019. Comparing year 2018 to 2019, the overall ENMR decreased significantly (OR 0.62; 95% confidence interval (CI) 0.45–0.85) as well as infants ≥37 weeks (OR 0.45) (CI 0.23–0.87) and infants < 37 weeks (OR 0.57) (CI 0.39–0.84). ENMR was significantly higher for newborns < 37 versus ≥37 weeks, OR 10.5 (*p* < 0.0001) and BW < 2500 versus ≥2500 g OR 9.9. For infants < 1000 g / < 28 weeks, the ENMR was ~ 588/1000 livebirths. Variables associated with ENM included BW - odds of death decreased by 0.55 for every 500 g increase in weight, by 0.89 for every week increase in GA, ENMR increased 6.8-fold with BMV, 2.6-fold with abnormal FHRT, 2.2-fold with no antenatal steroids (ANS), 2.6-fold with moderate hypothermia (all < 0.0001). The overall FSB rate was 14.7/1000 births and decreased significantly in 2019 when compared to 2015 i.e., 11.3 versus 17.3/1000 live births respectively (*p* = 0.02).

**Conclusion:**

ENM rates were predominantly modulated by decreasing BW and GA, with smaller/ less mature newborns 10-fold more likely to die. ENM in term newborns was strongly associated with FHRT abnormalities and when coupled with respiratory depression and BMV suggests BA. In smaller newborns, lack of ACS exposure and moderate hypothermia were additional associated factors. A composite perinatal approach is essential to achieve a sustained reduction in ENMR.

## Introduction

It is estimated that 2.7 million newborns die annually worldwide, which contributes to approximately 45% of under-5 child mortality [1, 2]. The first day and especially the first hour is critical to newborn survival, with the highest risk of intrapartum-related neonatal deaths (birth asphyxia (BA)) occurring during this period [1, 2]. In addition to BA (30 to 35%), prominent causes include prematurity/low birth weight (25 to 30%), presumed infection (~ 30%) and congenital anomalies (8–15%) [3]. An estimated 1.3 million babies are reported to be “fresh stillborn” (FSB), suggestive of an intrapartum demise, shortly before delivery [4, 5]. The Helping Babies Breathe (HBB) program was piloted in Tanzania in 2009, at a time where the neonatal mortality rate (NMR) approximated 25.3/1000 live births (LB). This study was associated with a 47% reduction in neonatal mortality (≤ 24 h) and a 24% reduction in FSB [6]. By 2015, more than 13,000 providers had been trained in HBB throughout Tanzania [7]. In 2015, a pilot study of a premature care bundle was implemented to mothers in preterm labor (28 to 34 weeks gestational age (GA)) and their newborns [8]. At the completion of the study in 2017, the care bundle was associated with a 26% reduction in ENM (< 7 days). By 2019, the overall NM (28-day mortality) in Tanzania had decreased to 20.3/1000 live births [9].

Kilimanjaro Christian Medical Center (KCMC) participated in both studies (HBB and the care bundle) and thus provides an opportunity to assess the impact of both interventions on ENM < 7 days over time. There are data to indicate that ENM is strongly influenced by GA and/or birth weight (BW) as well as variables during labor and the delivery room [10–23]. Many of the prior studies have not examined ENM rates over an extended period of time. Furthermore, the impact of a progressive decrease in both BW and GA on ENM remains unclear. This is relevant to KCMC, since a premature care bundle was introduced in that institution in 2015 and completed in 2017 [8]. The study objectives were: first, to determine the overall ENM as well as yearly ENM rate from 2015 to 2019; second, to determine the impact of decreasing GA (< 37 weeks) and BW (< 2500 g) on ENM; third, to determine the contribution of intrapartum and in particular fetal heart rate abnormalities (FHRT) and delivery room interventions on ENM; and fourth, to determine the FSB rates over the same time.

## Methods

This was a retrospective study of prospectively collected labor and delivery room data of newborns delivered at KCMC, a zonal referral University Teaching Hospital serving over 15 million people in Northern Tanzania for the period January 2015 to December 2019.

### Management of Mothers during labor using Fetal Heart Rate (FHRT)

During labor, FHRT is monitored by intermittent auscultation using a fetoscope, or intermittently/continuously with Doppler, which included Moyo (Laerdal Medical). Moyo is a novel Doppler machine that uses a 9-crystal sensor, which rapidly detects the FHRT [24]. Cardiotocography (CTG) is utilized for continuous external fetal monitoring (CEFM) in high-risk pregnancy cases. The midwife interprets the majority of the fetoscope and Doppler signals. The obstetrician interprets the CEFM. Fetal scalp blood gases or fetal stimulation is not done.

Indications for Cesarean Section (CS) include those considered absolute, i.e. contracted pelvis, placenta previa, and relative including abruption placentae with unfavorable cervix, fetal distress, and malpresentation.

The CS rate ranges between 34 to 44%. The approximate number of annual deliveries ranges from 4000 to 4500.

### Management of the Newborn in the delivery room

Midwives are the primary providers at the majority of spontaneous vaginal deliveries and are trained in HBB to manage resuscitation of the newborn. A self-inflation bag without positive end expiratory pressure is used for ventilation.

### Management of Newborns in the neonatal care area

High-risk newborns are admitted to a neonatal care unit with a capacity of 62 beds. The management of premature infants with respiratory distress includes continuous positive airway pressure (CPAP) (Pumani - Rice 360° Institute for Global Health Technologies) [25] where available; there are only two CPAP machines. Intubation and mechanical ventilation is not available. Additional interventions include intravenous antibiotics as indicated, and Kangaroo mother care to stable newborns.

#### Data monitoring

A dedicated computer close to the labor ward has been used for data entry since 2009. Data collection includes core and desired elements developed for the initial HBB rollout and expanded following implementation of the Care Bundle in 2015. Data retrieved included BW, GA, singletons/twins, fetal heart rate (FHRT) abnormalities on arrival, during, and prior to delivery (abnormal defined as < 120 or > 160 beats/minute), labor complications (including pre-eclampsia/eclampsia, malpresentation, arrest of descent), mode of delivery (vaginal, cesarean section, breech), bag mask ventilation (BMV)), 1 and 5 min Apgar scores, use of a care bundle (maternal and neonatal antibiotics where indicated, ANS to mothers of GA 28 to 34 weeks, maintaining infant temperature following delivery) [8]. Outcome was either survival or death ≤7 days. Data analysts (AS, PM) and a technical consultant (JMP) analyzed the data.

##### Definitions

BA was defined as a 5-minute Apgar score < 7 and lack of spontaneous respirations after birth [3]. GA was based on self-report of the last normal menstrual period and/or fundal height, the latter the distance from symphysis pubis to the uterine fundus in the middle of the woman’s abdomen, as is the standard practice in Tanzania [26]. Moderate preeclampsia was defined as a blood pressure > 140/90 mmHg with associated proteinuria and severe pre-eclampsia as a blood pressure ≥ 160/110 mmHg with specific signs and symptoms. Early neonatal mortality was death within the first 7 days following birth. Birth weight (BW) cutoff for live births was ≥750 g. Fresh stillbirth (FSB) was defined as an Apgar score = 0 at both 1 and 5 minutes with intact skin and suspected death during labour/delivery, and of birth weight > 1000 g. Perinatal mortality rate was defined as the number of early neonatal death < 7 days and FSB per 1000 live births.

#### Data analysis

Analysis has been performed using Statistical Package for Social Sciences (SPSS) 22; and included descriptive statistics, chi square analysis, t tests and odds ratio (OR) calculations. The outcome was early neonatal death < 7 days among live born neonates. A multiple regression model was developed to estimate the effects of BW, GA, referral versus inborn, gender, pre-eclampsia, multiples, mode of delivery (vaginal versus CS), abnormal FHRT on admission and prior to delivery, BMV, moderate hypothermia (initial temperature < 36 °C), ANS administration and ENM. Data was analyzed for the entire cohort followed by subgroup analysis for infants, < 37 weeks versus ≥37 weeks or < 2500 g versus ≥2500 g. All data are presented as mean ± standard deviation unless as otherwise stated.

##### Ethical considerations

This report reflects a retrospective review of data already collected. As such, no patient consent was obtained for this data review. The data had been prospectively obtained as part of implementation of a care bundle [8] which had received ethical clearance from the National Institute of Medical Research of Tanzania. (NIMR/HQ/R/R.8c/Vol.I/1156). These studies were performed in accordance with relevant guidelines and regulations. This study has been previously published (see reference [8]). Approval for extension of ethical clearance from the National Institute of Medical Research of Tanzania specifically for continued retrospective data review was subsequently obtained. (NIMR/HQ/R.8a/Vol.IX/1887).

## Results

### General

**Fig. 1:**
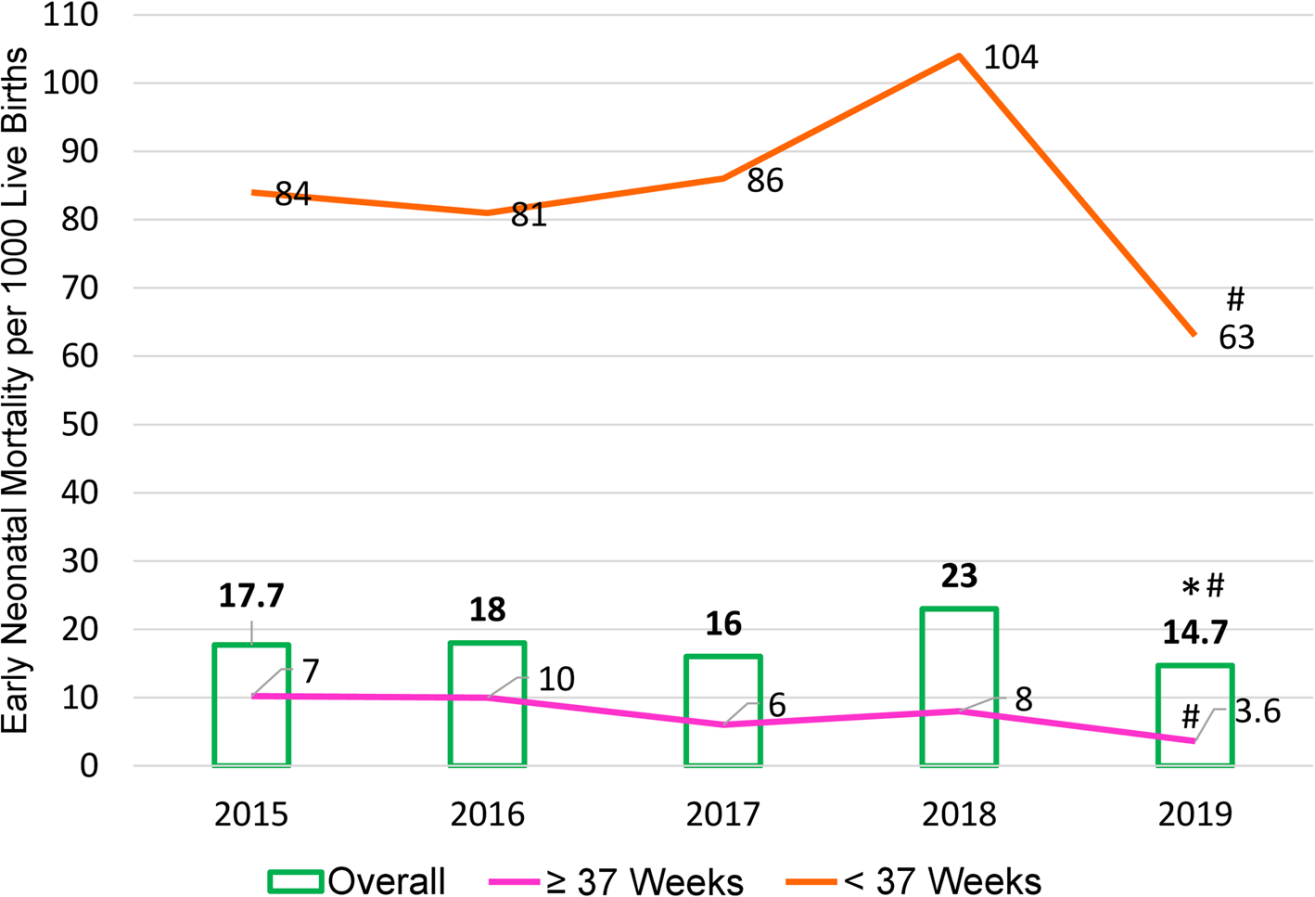
Overall Neonatal Mortality and for newborns < 37 and ≥ 37 weeks per 1000 live births for years 2015–2019. *Represent significant reduction in mortality for newborns ≥37 weeks when comparing 2015 and 2019. # Significant decreases in overall ENM when comparing 2019 versus 2018 as well as infants ≥37 weeks and < 37 weeks

Between January 2015 through December 2019 there were 20,760 deliveries, of which 20,250 (96%) were live births; 369 died (1.8%), 305 (1.5%) were FSB and 205 (1%) were macerated stillbirths. The overall ENMR was 18/1000 live births (range 14.7 to 23); for newborns < 37 weeks GA the ENMR was 81/1000 (range 63 to 104) and 8/1000 for newborns ≥37 weeks GA (range 3.6 to 10.2) (Fig. 1). The overall ENMR was comparable when comparing 2015 to subsequent years (Fig. 1). For newborns ≥37 weeks, ENMR’s were comparable for years 2015 through 2018 but less in 2019 (*p* = 0.0006). F205 or newborns < 37 weeks, ENMR’s were comparable for all years relative to 2015. ENMR was significantly higher for newborns < 37 versus ≥37 weeks, i.e., 81/1000 LB vs (8/1000 live births) (OR 10.5) (95% CI 8.5–13) respectively, and for BW < 2500 versus ≥2500 g, i.e., 75/1000 live births versus 7/1000 LB (OR 9.9) (95% CI 7.9 to 12.3) respectively.

Comparing year 2018 to 2019, the overall ENMR decreased significantly (OR 0.62; 95% confidence interval (CI) 0.45–0.85) (*p* = 0.0003). This decrease included infants ≥37 weeks (OR 0.45) (CI 0.23–0.87) (*p* = 0.01) and infants < 37 weeks (OR 0.57) (CI 0.39–0.84) (*p* = 0.004) (Fig. 1).

**Fig. 2:**
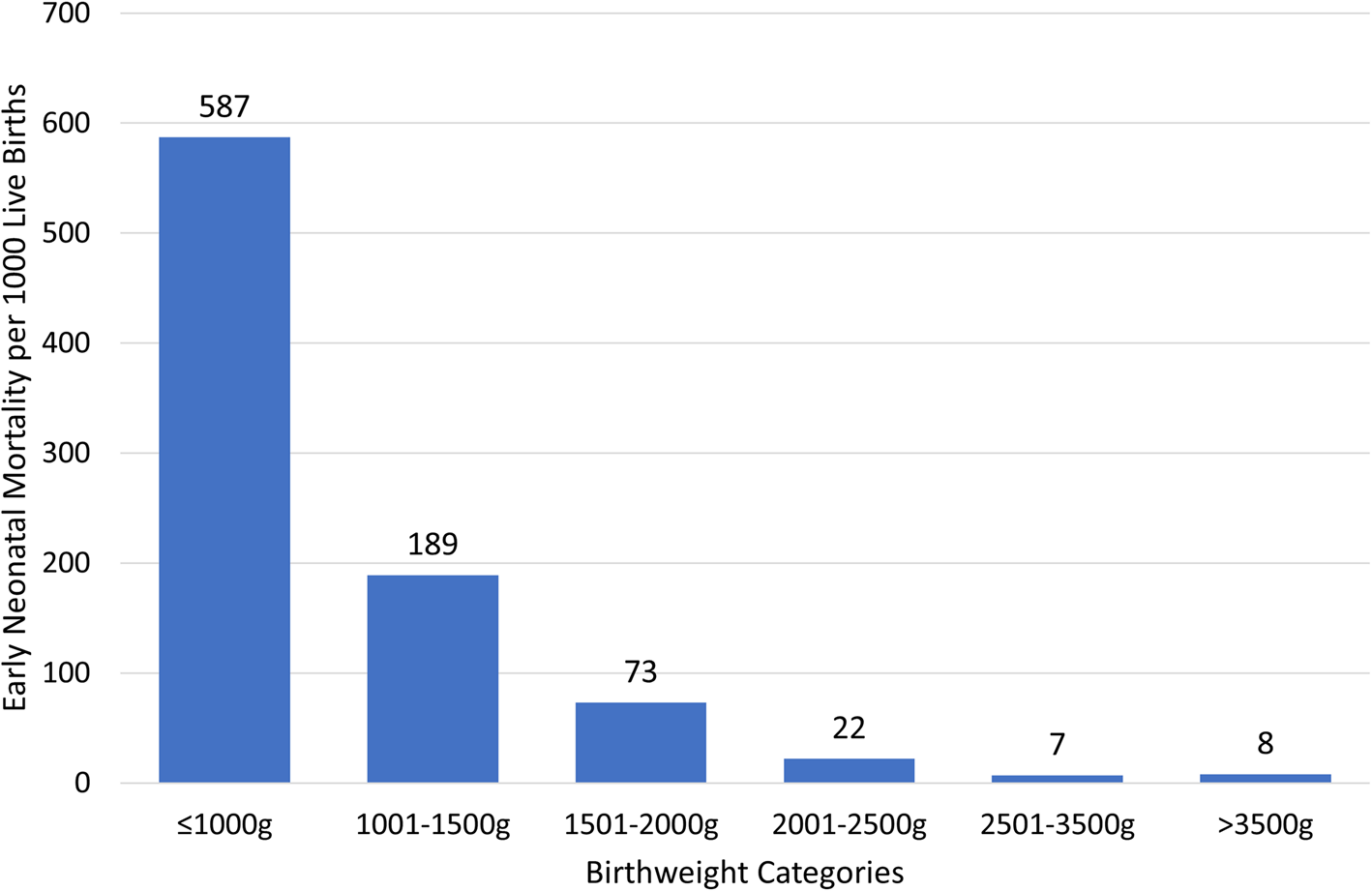
Early Neonatal Mortality Rate Per 1000 Live Births as a Function of Birth Weight

**Fig. 3:**
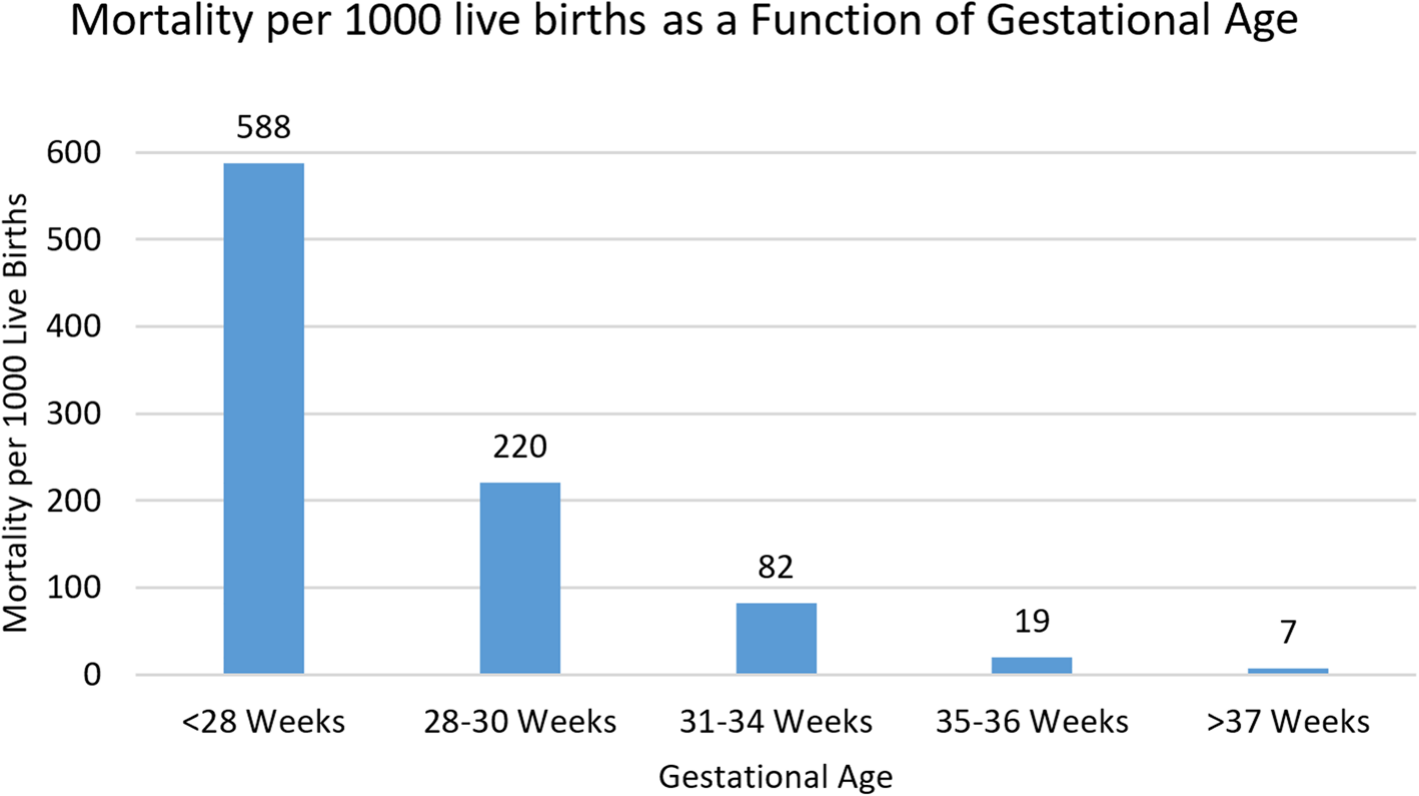
Early Neonatal Mortality per 1000 live births as a function of gestational age

The contribution of lesser BW and GA to overall neonatal mortality rate (NMR) is shown in Figs. 2 and 3. Specifically for infants < 1000 g or < 28 weeks, NMR was 587 and 588 per 1000 live births respectively. NMR progressively decreased with increasing BW and GA but was still substantial for BW 2000 to 2500 g at 22/1000 and 35 to 36 weeks at 19/1000. It was lowest for infants ≥2500 g or ≥ 37 weeks at 7 per 1000 live births (Figs. 2 and 3).

### Fresh stillbirth rates

The overall FSB rate was 305/20619 (14.7/1000 births). The overall FSB rate was comparable when comparing 2015 (17.2/1000) to subsequent years 2016 to 2018 (range 15.1 to 13.7/1000 births). The rate decreased significantly in 2019 compared to 2015 (11.3/1000 births (OR 0.65(0.45 to 0.93) (*p* = 0.02). When examined as a function of GA < 37 versus ≥37 weeks, FSB rates were 50 versus 8 per 1000 births (OR 6.1) (CI 4.9–7.7) (*p* < 0.0001 and < 2500 and ≥ 2500 g) the FSB rates were 45versus 8 per 1000 live births (OR 5.9) (CI 4.7–7.4) (*p* < 0.0001) respectively.

Perinatal Mortality rate did not differ when comparing 2015 (35.3 per 1000 LB) versus 2016 (32.2/1000 live births), 2017 (30.8//1000 live births) and 2018 (36.1/1000 live births but decreased significantly when compared to 2019 (26/1000 live births) (OR 0.65(0.45–0.93) (*p* = 0.0004).

### Characteristics of newborns who died compared to survivors

#### Entire cohort

Infants who died versus survivors were of a lesser BW (*p* < 0.0001) and GA (*p* < 0.0001). (Table 1) Infants who died versus survivors had a significantly lower initial temperature (*p* < 0.0001), were 1.3-fold more likely to be males (*p* = 0.009), 2.1-fold to be of a twin set (*p* < 0.0001), 1.68-fold to be associated with a maternal transfer (*p* < 0.0001), six-fold more likely to have any labor complications and specifically preeclampsia/eclampsia (*p* < 0.0001), 20-fold more likely to exhibit an abnormal FHRT on admission (*p* < 0.0001) and 33-fold more likely prior to delivery (*p* < 0.0001), 1.9-fold more likely be delivered via CS (*p* < 0.0001), 1.8-fold more likely to be delivered breech (*p* = 0.01), 161-fold more likely to have an Apgar score < 7 at 5 minutes (*p* < 0.0001), 116-fold more likely to receive BMV (*p* < 0.0001) and 11.9-fold more likely to exhibit moderate hypothermia (*p* < 0.0001).

**Table 1:** Perinatal characteristics of infants who survived versus those who died for the overall population 2015–2019

Characteristics	Survived *n* = 20,250	Died *n* = 369	*p* value	OR (95% CI)
Birth Weight (g)	3105 ± 612	2108 ± 954	< 0.0001	
Gestational Age (weeks)	38.4 ± 2.2	34.1 ± 4.5	< 0.0001	
Initial Temperature (°C)	36.3 ± 0.31	35.67 ± 0.37	< 0.0001	
Gender (Males)	11,020 (54%)	226 (61%)	0.009	1.3 (1.1–1.6)
Plurals	1008 (5%)	37 (10%)	< 0.0001	2.1 (1.5–3.9)
Maternal referral	8432 (42%)	201 (54%)	< 0.0001	1.7 (1.3–2.0)
Any Labor Complication	9816 (48.5%)	313 (85%)	< 0.0001	6.1 (4.5–8.1)
Eclampsia/Preeclampsia	712 (3.5%)	58 (15.7%)	< 0.0001	5.1(3.8–6.7)
Breech Presentation	585 (2.9%)	19 (5.1%)	0.01	1.8 (1.1–2.9)
Abnormal FHRT before Delivery	1303 (6.4%)	256 (69.8%)	< 0.0001	33.2 (26.4–41.8)
CS Delivery	8675 (44%)	220 (62%)	< 0.0001	1.9 (1.6–2.4)
Apgar at 1 minute	9 (10)	4 (9)	< 0.0001	
Apgar at 5 minutes	10 (10)	6 (10)	< 0.0001	
5 minute Apgar Score < 7	216 (1.1%)	234 (63.4%)	< 0.0001	161 (125–206)
Moderate Hypothermia (< 36 °C)	301/1195 (25%)	96/120 (80%)	< 0.0001	11.9 (7.4–18.8)
Bag/Mask Ventilation in DR	1734 (8.6%)	338 (91.6%)	< 0.0001	116 (80--168)

Apgar Score numbers are presented as Median and Interquartile range

*OR* Odds Ratio, *CI* Confidence Interval, *FHRT* Abnormal Fetal Heart Rate, *DR* Delivery Room

#### Multiple regression analysis

When controlling for other predictors, BW, GA, abnormal FHRT, and BMV contributed significantly to ENM. Specifically, for BW the odds of dying decreased 0.71 for each 500 g increase in BW, and it decreased 0.93 for each one-week increase in GA. The odds of dying increased four-fold with an abnormal FHRT prior to delivery (*p* < 0.0001), and 32-fold with receipt of BMV (*p* < 0.0001). There were too many missing temperature values for this variable to be included in the analysis.

### Characteristics of Newborns who Died compared to Survivors < 37 weeks EGA (Table 2)

Infants who died versus survivors were of a significantly lesser BW and GA (*p* < 0.0001), had a lower initial temperature (*p* < 0.0001), were 11.7-fold more likely to exhibit an abnormal FHRT prior to delivery (*p* < 0.0001), 3.4 fold to exhibit any labor complication and 1.8 fold to exhibit eclampsia/preeclampsia, 2.2-fold more likely to receive 3–4 doses as opposed to 0–2 doses of ANS (*p* < 0.0001), 42-fold more likely to be administered BMV (*p* < 0.0001), and 7.2-fold more likely to exhibit moderate hypothermia (*p* < 0.0001). Additional significant differences are shown in Table 2.

**Table 2:** Perinatal characteristics of infants who survived versus those who died < 37 weeks

Characteristics	Survived *n* = 2619	Died *n* = 232	*p* value	OR (95% CI)
Birth Weight (g)	2162 ± 560	1513 ± 560	< 0.0001	
Gestational Age (weeks)	33.93 ± 2.36	31.17 ± 2.85	< 0.0001	
Initial Temperature (°C)	36.03 ± 0.32	35.67 ± 0.37	< 0.0001	
Gender (Males)	1349 (51.4%)	142 (61.2%)	0.004	1.50 (1.13–1.96)
Plurals	408 (15.6%)	35 (15.1%)	0.85	
Referred	1202 (45.8%)	123 (53.0%)	0.03	1.3 (1.02–1.74
Any Labor Complications	1830 (69.9%)	205 (88.7%)	< 0.0001	3.4 (2.23–5.15)
Eclampsia/Preeclampsia	358 (13.7%)	51 (22.0%)	0.001	1.8 (1.28–2.48)
Breech	95 (3.6%)	14 (6%)	0.06	1.7 (0.96–3.04)
Abn FHRT before Delivery	491 (14.9%)	156 (67.2%)	< 0.0001	11.7 (8.72–15.72)
Cesarean Section Delivery	1397 (55.3%)	141 (63.5%)	0.01	1.4 (1.06–1.87)
Apgar Score at 1 min	8 (9)	4 (9)	< 0.0001	
Apgar Score at 5 min	10 (10)	6 (10)	< 0.0001	
Apgar Score at 5 minutes < 7	108 (4.1%)	140 (60.3%)	< 0.0001	35.4 (25.57–49.08)
Moderate Hypothermia (< 36 °C)	292/1148 (25.4%)	94/118 (79.7%)	< 0.0001	7.2 (11.49–18.18)
Bag mask ventilation in DR	741 (28.3%)	218 (94.4%)	< 0.0001	42.5 (24.14–74.89)
ANS 0–2 vs, 3–4 doses	1815 (69.2%)	193 (83.2%)	< 0.0001	2.20 (1.54–3.14)

Apgar score numbers are presented as Median and Interquartile range

*Abn FHRT* Abnormal Fetal Heart Rate, *DR* Delivery Room, *ANS* Antenatal steroids

#### Multiple regression analysis

Five variables were significantly associated with ENM. When controlling for other predictors, the odds of death decreased by 0.55 for every 500 g increase in BW and by 0.89-fold for every 1 week increase in GA. The odds of dying increased 6.8-fold with BMV application (*p* < 0.0001), 2.6-fold with an abnormal FHRT prior to delivery (*p* < 0.0001), and 2.6-fold with moderate hypothermia (< 0.0001).

### Characteristics of newborns who died compared to survivors ≥37 weeks GA (Table 3)

Infants ≥37 weeks GA who died versus those who survived were of significant lesser BW (*p* < 0.0001) but comparable GA (*p* = 0.58). Infants were 50-fold more likely to have an abnormal FHRT prior to delivery (*p* < 0.0001), 4.4 fold to exhibit any labor complication and 2.6 fold to exhibit eclampsia/preeclampsia, 118-fold more likely to be administered BMV and 350-fold more likely to have a 5 minute Apgar score < 7 at 5 minutes. There were too few newborns in this GA group with a temperature measurement to include in the analysis.

**Table 3:** Perinatal characteristics of infants who survived versus those who died ≥37 weeks

Characteristics	Survived *n* = 17,617	Died *n* = 138	*p* value	OR (95% CI)
Birth Weight (g)	3245 ± 482	3102 ± 571	0.001	
Gestational Age (weeks)	39.15 ± 1.08	39.09 ± 1.33	0.58	
Male	9669 (54.9%)	84 (61.3%)	0.13	1.3 (0.92–1.84)
Plurals	600 (3.4%)	2/135 (1.5%)	0.33	
Referred	7230 (41.1%)	78 (56.9%)	0.002	1.9 (1.35–2.67)
Any Labor Complications	7964 (45.3%)	108 (78.8%)	< 0.0001	4.4 (2.97–6.75)
Eclampsia/Preeclampsia	363 (2.0%)	7 (5.1%)	0.02	2.6 (1.22–5.62)
Breech	490 (2.8%)	5 (3.6%)	0.53	
Abnormal FHRT before Delivery	912 (5.2%)	100 (73.5%)	< 0.0001	50.8 (34.6–74.9)
Cesarean Section Delivery	7276 (41.9%)	79 (60.3%)	< 0.0001	2.1 (1.48–2.99)
Apgar at 1 min	9 (10)	4 (10)	< 0.0001	
Apgar at 5 min	10 (10)	6 (10)	< 0.0001	
Apgar Score at 5 min < 7	108 (0.8%)	94 (66.6%)	< 0.0001	354 (235–532)
Bag Mask Ventilation in the DR	992 (5.6%)	120 (87.6%)	< 0.0001	118 (71–197)

Hypothermia not included because of very small numbers

Apgar score numbers presented as Median and Interquartile range

*FHRT* Fetal Heart Rate, *DR* Delivery Room

#### Multiple regression analysis

Only an abnormal FHRT prior to delivery and BMV were significantly associated with ENM. The odds of dying increased 7.3-fold with an abnormal FHRT (*p* < 0.0001) and 42-fold (*p* < 0.0001) with BMV.

### Outcome as a function of birth weight < 2500 g (Table 4)

Infants who died versus those who survived were of a lesser BW (*p* < 0.0001) and GA (*p* < 0.0001), had a lower initial temperature (*p* < 0.0001), 3.2 fold more likely to exhibit any labor complication and 1.9 fold to exhibit eclampsia/preeclampsia, were 1.7-fold more likely to receive 3 to 4 doses as opposed to 0 to 2 of ANS (*p* = 0.002), 13.6-fold more likely to exhibit an abnormal FHRT prior to delivery (*p* < 0.001), 48.7-fold more likely to be administered BMV (*p* < 0.001), and 11.3-fold more likely to exhibit moderate hypothermia (*p* < 0.0001) (Table 4).

**Table 4:** Perinatal characteristics of infants who survived versus those who died ≤2500 g

Characteristics	Survived *n* = 3187	Died *n* = 241	*p* value	OR (95% CI)
Birth Weight (grams)	2072 ± 385	1507 ± 504	< 0.0001	
Gestational Age (weeks)	35.18 ± 3.02	31.59 ± 3.3	< 0.0001	
Initial Temperature (°C)	36.03 ± 0.31	35.67 ± 0.37	< 0.0001	
Males	1562 (49%)	147 (61%)	0.0003	1.6 (1.24–2.12)
Plurals	681 (21.4%)	36 (14.9%)	0.01	0.6 (0.4–0.93)
Referred	1516 (47.6%)	128 (53.1%)	0.09	1.2 (0.96–1.62)
Any Labor Complications	2178 (68.5%)	210 (87.5%)	< 0.0001	3.2 (2.18–4.76)
Eclampsia/Preeclampsia	394 (12.4%)	52 (21.6%)	< 0.0001	1.9 (1.41–2.70)
Breech	118 (3.7%)	14 (5.8%)	0.10	1.6 (0.91–2.84)
Abnormal FHRT before Delivery	432 (13.6%)	164 (68%)	< 0.0001	13.6 (10.2–18.1)
Cesarean Section Delivery	1640 (53.5%)	140 (60.9%)	0.03	1.3 (1.03–1.78)
Apgar at 1 min	8 (9)	4 (9)	< 0.0001	
Apgar at 5 min	10 (10)	6 (10)	< 0.0001	
5 Minute Apgar Score < 7	110 (3.5%)	147 (61.0%)	< 0.0001	43.7 (31.7–60.3)
Moderate Hypothermia < 36 °C	294/1138 (25%)	95/119 (79%)	< 0.0001	11.3 (7.1–18.2)
Bag/Mask Ventilation in DR	793 (25%)	226 (94%)	< 0.0001	48.7 (28.5–84)
ANS 0–2 doses	2394 (75%)	202 (83%)	0.002	1.72 (1.2–2.4)

Apgar score numbers are Median and Interquartile range

*FHRT* Fetal Heart Rate, *DR* Delivery Room, *ANS* antenatal steroids

#### Multiple regression analysis

When controlling for other predictors, six variables were significantly associated with NM. These included BW, where the odds of dying decreased by 0.57 for every 500 g increase in weight and by 0.86 for every one-week increase in GA. The odds of dying was 1.6-fold higher in male infants (*p* = 0.004), increased 2.7-fold with an abnormal FHRT prior to delivery (*p* < 0.0001) and 14.7-fold in infants administered BMV (*p* < 0.0001).

### Newborns > 2500 g (Table 5)

Infants who died versus those who survived were of comparable BW but lesser GA (*p* = 0.04), were 26-fold more likely to have an abnormal FHRT upon admission (*p* < 0.0001), 48.8-fold more likely exhibit an abnormal FHRT prior to delivery (*p* < 0.0001), 5.0 fold to exhibit any labor complication and 2.59 fold to exhibit eclampsia/preeclampsia, and 119-fold more likely be more likely to be administered BMV in the DR.

**Table 5:** Perinatal characteristics associated with outcome for newborns > 2500 g

Characteristics	Survived *n* = 17,058	Died *n* = 128	*p* value	OR (95% CI)
Birth Weight (g)	3298 ± 426	3238 ± 433	0.11	
Gestational Age (weeks)	39.08 ± 0.42	38.86 ± 1.94	0.04	
Males	9454 (55.4%)	49 (61.7%)	0.15	1.29 (0.91–1.86)
Plurals	327 (1.9%)	1 (0.8%)	0.36	0.40 (0.05–2.89)
Referred	6913 (41%)	73 (57%)	0.0002	1.95 (1.37–2.76)
Any Labor Complications	7634 (44.8%)	103 (80.5%)	< 0.0001	5.08 (3.27–7.87)
Eclampsia/Preeclampsia	317 (1.9%)	6 (4.6%)	0.03	2.59 (1.13–5.95)
Breech	467 (2.7%)	5 (3.9%)	0.40	1.44 (0.58–3.55)
Abnormal FHRT before Delivery	871 (5.1%)	92/127 (72.4%)	< 0.0001	48.8 (32.9–72.52)
Cesarean Section Delivery	7032 (41.8%)	80 (65%)	< 0.0001	2.59 (1.78–3.75)
Apgar at 1 min	9 (10)	4 (9)	< 0.0001	
Apgar at 5 min	10 (10)	5 (10)	< 0.0001	
5 Minute Apgar Score < 7	106 (0.6%)	87 (68%)	< 0.0001	339 (223–515)
Bag/Mask Ventilation in the DR	941 (5.5%)	112 (87.5%)	< 0.0001	119 (71–203)

Hypothermia not included because of small numbers

Apgar score numbers are Median and Interquartile range

*FHRT* Fetal Heart Rate, *DR* Delivery Room

#### Multiple regression analysis (> 2500 g)

When holding other variables constant only BMV and FTHR prior to delivery had a significant effect on ENM. The odds of dying were 44.8-fold higher (*p* < 0.0001) for those newborns resuscitated with BMV and 7.0-fold higher (*p* < 0.0001) with an abnormal FHRT prior to delivery.

## Discussion

The findings in this report indicate that the overall ENMR for the 5 years was approximately 18/1000 live births ranging from 14 to 23/1000 live births. ENM was strongly influenced by both BW and GA. Thus, for newborns < 2500 g BW, ENMR was 75/1000 live births and 7/1000 live births for those ≥2500 g i.e., 10-fold more likely to die. For newborns < 37 weeks, ENMR was approximately 81/1000 live births (range 63 to 104) and ≥ 37 weeks 8/1000 live births (range 3 to 10.2) i.e., 10-fold more likely to die. Furthermore, ENM increased markedly as a function of decreasing BW and/or GA. Thus, the highest ENMR was for infants ≤1000 g BW or ≤ 28 weeks GA with a rate of approximately 588/1000 live births i.e., ~ 75-fold more likely to die as opposed to neonates > 2500 g and/or > 37 weeks GA. For the entire cohort, the odds of dying deceased 29% for every 500 g increase in BW, and 11% for each week increase in GA. Additional contributors to ENM included an abnormal FHRT prior to delivery, as well as the application of BMV during delivery room resuscitation. For the smaller newborns moderate hypothermia also significantly increased the likelihood of ENM.

When examined over time, the overall ENMR as well as that of newborns < 37 weeks GA demonstrated no significant year-to-year differences when comparing 2015 to subsequent years. An exception was a significant decrease in ENM noted in 2019 when compared to 2015. Interestingly when comparing year 2019 to 2018, the overall ENM, as well as that of newborns ≥37 and < 37 weeks decreased significantly (Fig. 1). We speculate that the reduction in neonates < 37 weeks may have reflected full implementation of the care bundle [8]. The above observations are important in that when describing ENMR’s, these should be viewed over time, as well as a function of BW/GA. Notably the highest ENMR was in the smallest newborns, i.e., ≤ 1000 g or < 28 weeks and was close to 60%. These latter observations are similar to prior reports demonstrating extremely high mortality in the tiniest premature infants [17–23]. Moreover in a recent large prospective population-based study undertaken in a low resource setting, BW was noted to be to the most important variable for predicting the risk of neonatal mortality [17].

The wide disparity in ENM expressed as either BW or GA offers an opportunity for more targeted interventions. For infants > 2500 g and /or > 37 weeks, the pathway to death appears to be mediated or initiated via intrapartum factors identified by an abnormal FHRT prior to delivery. This finding when coupled with respiratory depression at birth, as indicated by a 5-minute Apgar score < 7 and an increased requirement for BMV, is consistent with the World Health Organization definition of BA [3, 27, 28]. For those newborns < 2500 g and /or < 37 weeks, in addition to an abnormal FHRT and BMV, not receiving ANS, and presenting with initial moderate hypothermia were risk factors associated with increased likelihood of ENM. In a recent population based study two factors that increased the risk for neonatal death were prematurity and a poor condition at 5 minutes [29]. Potential pathways contributing to death in these infants in the low resource setting include respiratory distress particularly in the absence of ACS [30] and exacerbated by moderate hypothermia, which has been shown to be an independent risk factor for mortality [31–33]. In addition there was limited respiratory support; only two CPAP machines were available during the 5 years.

These findings indicate that strategies to reduce ENM need to be initiated upon arrival in the delivery room with a major focus on FHRT monitoring. At KCMC, the predominant method of detecting an abnormal FHRT is via the intermittent use of a fetoscope, less often using Doppler. Recently Moyo, a novel Doppler machine, has been used more frequently to monitor FHRT [24]. This may have contributed to the reduction in ENM in 2019 in infants ≥37 weeks. Recognition of an abnormal FHRT and prompt intervention may be of particular importance in reducing ENM. ANS administration to mothers of GA 28 to 34 weeks has been shown to reduce ENM in the low resource setting [30]. Upon delivery instituting the steps contained in the HBB within the Golden Minute and instituting interventions as needed, including BMV and avoiding hypothermia with early initiation of KMC are essential strategies [34]. Procurement of additional CPAP machines is important to treat respiratory distress, as needed [35].Additional training of physicians and nurses who specialize in the management of very preterm babies and use these more advanced strategies is essential.

The overall occurrence of FSB was 14.7/1000 births and was comparable for years 2015 through 2018 but decreased significantly in 2019. The reason for this significant reduction in 2019 remains unclear. The perinatal mortality rate did not differ when comparing 2015 (35.3 per 1000 live births) through 2018 (36.1/1000 live births) but decreased significantly in 2019 (26/1000 live births). The initial perinatal mortality rate is slightly lower than that reported from the same institution for years 2010 through 2015 [36].

The study has several limitations. First, we did not examine the contribution of antenatal factors, which have been found in other studies to contribute to ENM [10, 11, 13, 14]. In this regard, it is notable that a maternal referral was associated with a 1.7-fold increased likelihood of death. Second, the report represents a single center, and the findings may not be generalizable to other regions of Tanzania, or other low resource countries. Third, the categorization of FHRT as abnormal does not describe the specific abnormality. Fourth, any labor complications (such as malpresentation, arrest of descent) as a grouping was significantly associated with ENM. However, other than for pre-eclampsia, these complications were relatively infrequent to be able to demonstrate significant individual differences in outcome. Fifth, it was not possible to accurately identify the contribution of small for gestational age to overall ENM. Sixth, the putative causes of death was not available. Seventh, we do not have 28-day follow-up.

In conclusion, these data indicate that ENM is predominantly modulated by decreasing BW and GA, with smaller and less mature newborn 10-fold more likely to die as compared to the larger more mature newborn. The pathway to death in the term newborn appears to be triggered by intrapartum factors specifically FHRT. The presence of FHRT abnormalities coupled with respiratory depression and BMV at birth suggests a diagnosis of BA. In smaller babies in addition to the above, a lack of ANS exposure and moderate hypothermia appear to be additional contributory factors. To achieve a sustained reduction in NM, a composite perinatal approach is essential initiated upon admission to the delivery suite.

## Data Availability

The data that support the findings of this study are available from Dr. Hussein Kidanto but restrictions apply to the availability of these data, which were used under license for the current study, and so are not publicly available. Data are however available from the authors upon reasonable request and with permission of Dr. Hussein Kidanto (hkidanto@gmail.com).

## Abbreviations

BA: Birth Asphyxi
FSB: Fresh Still Birth
HBB: Helping Babies Breathe
ENM: Early Neonatal Mortality
ENMR: Early Neonatal Mortality Rate
NMR: Neonatal Mortality Rate
GA: Gestational Age
BW: Birth Weight
KCMC: Kilimanjaro Christian Medical Centre
CEFM: Continuous electronic fetal monitoring
FHRT: Fetal Heart Rate
CS: Cesarean Section
CPAP: Continuous Positive Airway Pressure
ACS: Antenatal Corticosteroids
SPSS: Statistical Package of Social Sciences
OR: Odds Ratio
CI: Confidence Interval
BMV: Bag Mask Ventilation
